# Current News and Opinion

**Published:** 1884-06

**Authors:** 


### Current News and Opinion.

### WESTERN MISCELLANY.

The thirteenth annual meeting of the Kansas State Dental Society was held at Hiawatha, Kansas, commencing May 6th, and continued three days, in joint session with the Nebraska State Society.

Both societies were well represented, and the meetings were full of interest. The officers elect for the Kansas Association are as follows :

President—Dr. J. A. Young, Emporia.

First Vice-President—Dr. W. M. Shirley, Hiawatha.

Second Vice-President—Dr. A. C. Schell, Kansas City.

Secretary—Dr. C. B. Reed, Topeka.

Treasurer—Dr. A. H. Thompson, Topeka.

The next meeting will be held at Topeka, Kansas, commencing the first Tuesday in May, 1885.

Dr. J. W. Chaddick, of Nebraska City, was elected President of the Nebraska Society. Your correspondent failed to get the names of the other officers elected.

The next meeting of the Nebraska Society will be held at Lincoln, commencing the third Tuesday in May, 1885.

Dr. L. P. Meredith, of Abiline, Kansas, is no more. No particulars concerning his death can now be obtained, further than that it was the result of a severe cold, contracted while out on a hunting expedition.

Dr. Meredith was in many ways a very remarkable man. His fine personal appearance and modest but dignified manner, coupled with scholarly attainments, made him many friends and admirers wherever he went. As a writer upon dental and other subjects he was well known to a majority of the profession.

He formerly lived at Cincinnati, O., where he enjoyed a very lucrative practice, and was regarded as a very excellent operator, but his health failing, he went west, locating at Abiline, Kansas, where he for a time engaged in the book and stationery business, paying but little attention to dentistry, except to keep himself posted through the medium of its journals, and occasionally to perform an operation for some personal friend.

Although not actively identified with the practice of dentistry, he never lost interest in its progress, and since his removal to Kansas had been a regular and faithful attendant upon the meetings of the State Association, of which he was President during the year 1882-3. A good man has gone.

PERSONAL.

Dr. J. D. Patterson, for the past fourteen years located at Lawrence, Kansas, has removed to Kansas City.

Dr. D. J. McMillen, formerly of Brunswick, Mo., and the present President of the Missouri State Association, has also located at Kansas City. "DENTOS."

---

WHAT DOES IT MEAN?

The last number of the *Odontographic Journal*, on its congratulatory page, after referring to its steady advancement from a comparatively obscure publication to that of a first-class and popular dental quarterly, and crediting its success to the energy, ability and good management of its editor and conductor, makes the following extraordinary statement: "The reading matter of the journal will, as heretofore, be in charge of the present conductor, who will say and permit others to say in its pages just what in his judgment should be said, with the consent of the publishers."

A curious sort of combination this of trust and censorship, and a strange show of consistency. An editor and "conductor" whose faultless "management" is acknowledged in fitting terms as almost the sole cause of the success of a journal, is permitted to exercise his individual judgment concerning its reading matter *with the consent of the publishers!* Is this another "hard hit"? Perhaps in these precarious times the publishers feel that it is not prudent to give their editor too much play of his *Line*, lest things get entangled. F.

---

A. D. S. E.

The American Dental Society of Europe will hold its next annual meeting at Vevey, Switzerland, beginning Tuesday, August 18th. Members of the profession are cordially invited to be present.

W. D. MILLER, Chairman of Ex. Com.

### MISSOURI.

The twentieth annual meeting of the Missouri State Dental Association will be held at Sweet Springs, Saline Co., Mo., commencing Tuesday, July 8th, and continuing four days. Reduced railroad and hotel rates have been secured, and a large attendance is expected. Every member of the profession is cordially invited to attend. For programmes and other information address Dr. G. W. Tindall, Chairman Executive Committee, 1108 Main Street, Kansas City, Mo.

G. W. TINDALL,

S. B. PREVOST,

E. E. SHATTUCK, } *Committee.*

### SIXTH DISTRICT DENTAL SOCIETY OF NEW YORK.

The Sixth District Dental Society held its fifteenth annual meeting at Binghamton, on Tuesday, May 6th. The following officers were elected:

President—M. D. Jewell, Richfield Springs.

Vice-President—C. E. Dunton, Cazenovia.

Rec. and Cor. Secretary—E. D. Downs, Owego.

Treasurer—Frank B. Darby, Elmira.

Censor—A. M. Holmes, Morrisville.

Delegates to State Society for four years—M. D. Jewell, Richfield Springs, E. D. Downs, Owego. E. D. DOWNS, D. D. S., Sec'y.

### MASSACHUSETTS DENTAL SOCIETY.

The nineteenth semi-annual meeting of the Massachusetts Dental Society will be held at the Hawthorne Rooms, 2 Park Street, Boston, Mass., on Thursday and Friday, June 5th and 6th, 1884, commencing at 11 o'clock Thursday morning.

W. E. PAGE, Secretary.

### CHANGE OF DATE.

As the dedication of the Buckingham Monument takes place at Hartford, June 18th, the meeting of the Connecticut Valley Dental Society has been changed from June 18th and 19th to June 19th and 20th, at Savin Rock, New Haven, Conn.

W. F. ANDREWS, Secretary.

(6 ) Will some one kindly suggest, through your columns, a remedy for the following case?

Mr. L——, a middle-aged man possessing usual health, has the habit of grinding his teeth during sleep only. The vitality of two of them has already been destroyed, and much injury done to others by breaking down strong walls from fillings. T. A. K.

(7.) A professional friend recently suggested that I line the cervical walls of large approximal cavities in molars and bicuspids with Robinson's fibrous tin. I have tried it and find that it works beautifully. Is there any objection to placing the two metals together in the same cavity, on account of galvanic or chemical action? S. D. C.

(8.) A statement was once made by a gentleman connected with one of the dental societies in New York, that "it is impossible to devitalize a tooth-pulp by the use of arsenious acid in any case where camphor has been previously applied." Will the party who made this statement please give his experience in this direction? D. F. M.

(9.) What peculiarity does the so-called "Electric" gold possess, and why so called? L.

(10.) I lately noticed in a medical journal a recommendation of the method of administering an anæsthetic *per rectum*. Is it possible to produce the effects in that manner, and, if so, what is the modus operandi? INQUIRER.

*Answers.*—J. L. D. (No. 1.) will find the answer to his question upon page 180 of the April No. of this Journal. I believe that the cause of the acute periostitis in such cases arises from the admission of air containing septic organisms into the pulp chamber. The remedy is in the so-called "Listerian" system. Open the pulp chamber under aseptic conditions. Prevent the entrance of septic germs, and the after treatment is easy. C. F. W. B.

Tell J. S. (No. 4.) to try *antimonium crudum*, 30th trituration. Sometimes *mercurius vivus* is preferable. C. W. S., St. Louis.

